# Earthworm-associated microbial community reassembly accompanies higher inorganic-N concentrations in spent mushroom substrate

**DOI:** 10.3389/fmicb.2026.1935901

**Published:** 2026-09-10

**Authors:** Ziming Ren, Chunyang Du, Yue Wang, Shujuan Ji, Xiangdan Chi, Yukun Ma, Zhu Lu, Xiao Li, Bo Zhang, Chao Shi

**Affiliations:** 1 Sanjiang Laboratory, Changchun, China; 2 Jilin Province Vegetable and Flower Research Institute, Changchun, China; 3 Engineering Research Center of Edible and Medicinal Fungi, Ministry of Education, Jilin Agricultural University, Changchun, China; 4 College of Mycology, Jilin Agricultural University, Changchun, China; 5 Industrial Development Institute for Plants, Animals and Fungi Integration of Biyang County, Zhumadian, China; 6 Hinggan League Institute of Agricultural and Husbandry Sciences, Inner Mongolia Innovation Center of Biological Breeding Technology, Ulan Hot City, China

**Keywords:** spent mushroom substrate, earthworm composting, *Eisenia fetida*, nitrogen transformation, bacterial community, inorganic nitrogen, PICRUSt2, microbial network

## Abstract

**Introduction:**

Spent mushroom substrate (SMS) is a major residual stream from edible mushroom production and a potential feedstock for organic fertilizer. Although earthworm composting can alter SMS transformation, the microbial processes associated with nitrogen transformation remain poorly resolved.

**Methods:**

Seven mixtures of black fungus SMS (AhSMS) and golden needle mushroom SMS (FfSMS) were subjected to conventional composting or *Eisenia fetida*-mediated composting and sampled after 0, 30, and 60 d.

**Results:**

Earthworm composting was associated with higher bacterial alpha-diversity values and a transition from early Bacteroidota-dominated communities to later communities enriched in Pseudomonadota and Actinomycetota. At the treatment level, mean NH4^+^-N in Group E increased from 2.34 to 44.17 mg kg^−1^ between day 0 and day 60 (1788%), while NO3^−^-N increased from 10.33 to 26.10 mg kg^−1^ (153%). These changes coincided with candidate taxa associated with predicted nitrogen-cycling functions, including *Arenimonas*, *Pseudoxanthomonas*, *Croceibacterium* and *Kineobacterium*. PICRUSt2-based functional prediction and network analysis further identified coordinated changes in predicted nitrogen-cycle EC categories and candidate hub taxa, including *Mesorhizobium* and *Flavobacterium*.

**Discussion:**

Earthworm composting was associated with higher inorganic-N concentrations and bacterial community reassembly. However, these concentration changes do not by themselves demonstrate greater total nitrogen retention because dry-matter loss was not measured. Moreover, as the functional profiles were inferred from 16S rRNA gene data, metagenomic and transcriptomic analyses are required to verify the underlying mechanisms. These findings provide a framework for optimizing earthworm-assisted SMS valorization and prioritizing microbial taxa and nitrogen-cycling pathways for functional validation.

## Graphical abstract

**Figure:**
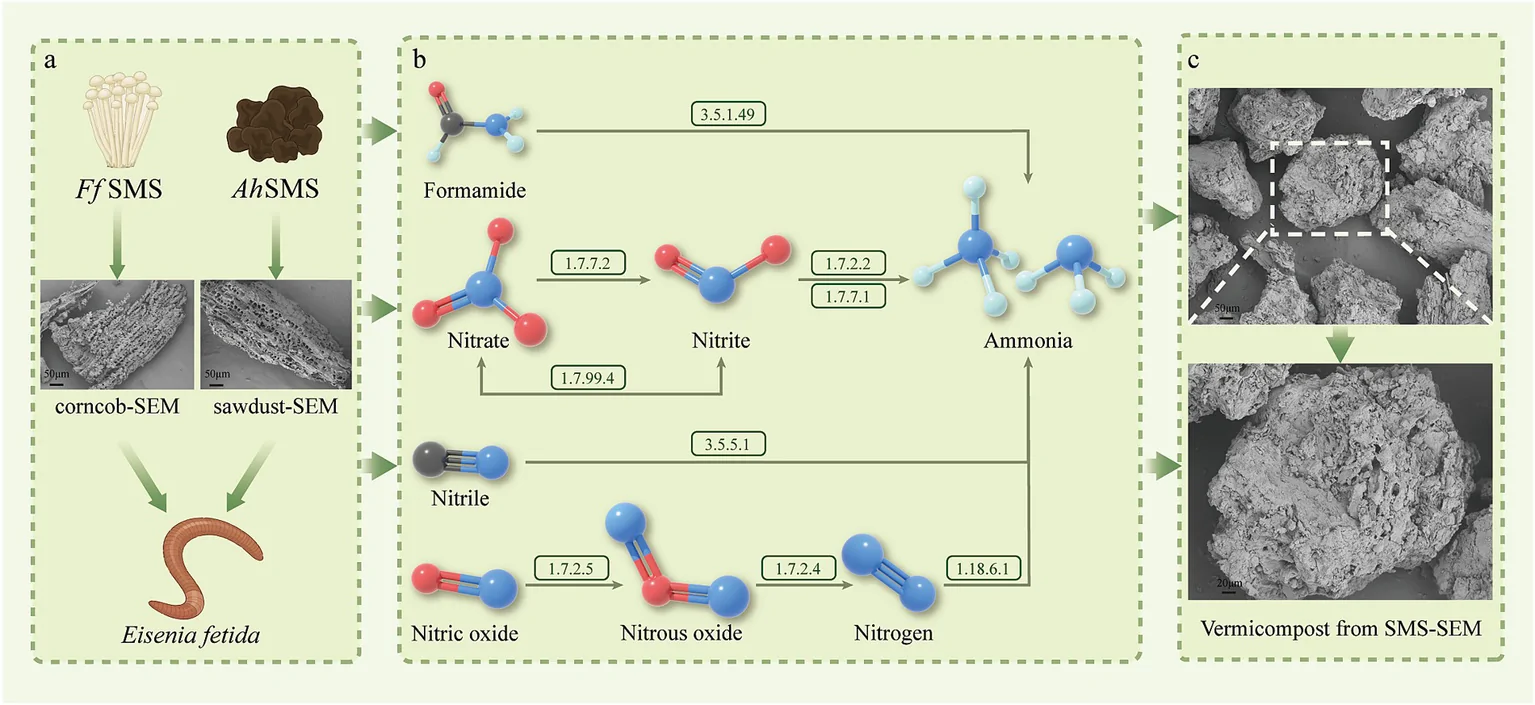

## 1 Introduction

Expansion of edible mushroom production has increased the volume of spent mushroom substrate (SMS), the lignocellulosic residue remaining after fruiting-body harvest. Managing SMS imposes storage, transport and disposal costs in mushroom-producing regions. However, SMS retains cellulose, hemicellulose, lignin, fungal biomass, organic matter and plant nutrients, making it a potential feedstock for soil amendments and organic fertilizers in a circular bioeconomy (Kousar et al., 2024; Martín et al., 2023). Its variable composition, incomplete stabilization and recalcitrant lignocellulose can hinder direct agricultural use. Biological treatment is therefore needed to promote substrate decomposition and nutrient release (Long et al., 2024; Sun et al., 2021; Yu et al., 2022).

Nitrogen transformation is particularly important because total nitrogen alone does not determine fertilizer value. During composting, polymer-bound organic nitrogen is depolymerized and ammonified to NH_4_^+_^N, which may subsequently be immobilized, nitrified to NO_3_^−_^N or lost through gaseous pathways. This balance depends on the carbon-to-nitrogen ratio, oxygen status, moisture, pH, extracellular enzyme activity and microbial community composition (Kuypers et al., 2018; Lv et al., 2019; Wang et al., 2022). Microbial succession can therefore influence the partitioning of nitrogen between plant-available and loss pools. In composting systems, shifts in community composition and co-occurrence structure often accompany changes in nitrogen-transforming genera, highlighting the value of integrating chemical and microbial-ecological analyses (Bello et al., 2020).

Vermicomposting combines microbial decomposition with the physical and biological activities of earthworms. Feeding, grinding, mixing and casting can fragment substrates, redistribute microorganisms and modify microscale nutrient availability and aeration. Gut passage and fresh casts may impose additional selective pressures while creating nutrient-rich microsites for microbial growth. These processes can promote stabilization and alter carbon and nitrogen transformation. In SMS-based systems, co-composting and vermicomposting can improve product maturity and nutrient quality (Gong et al., 2019; Yu et al., 2022), while earthworms can remodel prokaryotic communities and extracellular enzyme activities in soil microcosms (Buivydaitė et al., 2023). Across diverse organic residues, vermicomposting often improves residue stabilization and fertilizer-related properties, although outcomes vary with feedstock and operating conditions (Akhila and Entoori, 2022; Singh et al., 2020; Vuković et al., 2021). Product quality also depends on substrate composition and process configuration (Berhe et al., 2025; Dejene et al., 2024; Hidayati et al., 2021). Carbon-rich additives, closed-reactor designs and staged processing can further influence microbial activity, humification and vermicompost quality (Cao et al., 2021; Ghorbani et al., 2021; Kauser and Khwairakpam, 2022).

Earthworm density and substrate characteristics can affect microbial community assembly and nutrient transformations during vermicomposting (Devi et al., 2023; Liu X. et al., 2021; Wang et al., 2021). Earthworm-associated shifts in bacterial composition and extracellular enzyme activity have also been reported across plant- and residue-based systems (Lu et al., 2021; Turp et al., 2023; Wu et al., 2024). Extracellular enzyme dynamics are sensitive to substrate availability and environmental context during organic-matter transformation (Alves et al., 2021; Lou et al., 2022; Mohite et al., 2024). Compost amendments and long-term organic inputs can restructure soil microbial communities and nitrogen-related ecological interactions (Awasthi et al., 2020; Cuartero et al., 2022; Liu J. et al., 2021). More broadly, microbial diversity and plant-associated microbiome assembly respond to environmental management and habitat context (Jansson and Hofmockel, 2020; Trivedi et al., 2020; Walters and Martiny, 2020). However, many vermicomposting studies still focus on endpoint properties or final vermicompost quality, with limited temporal resolution of microbial processes involved in nitrogen mineralization.

A key unresolved question is how earthworm-associated bacterial community reassembly relates to nitrogen transformation across SMS compositions and composting stages. Metataxonomic sequencing can characterize community succession, and Phylogenetic Investigation of Communities by Reconstruction of Unobserved States version 2 (PICRUSt2) can infer potential functional profiles from 16S rRNA gene data (Douglas et al., 2020). Co-occurrence networks can identify taxa with coordinated abundance patterns, whereas multivariable association approaches can help distinguish candidate taxon–environment relationships from compositional and covariate effects (Mallick et al., 2021). Nevertheless, neither predicted functions nor statistical associations can demonstrate gene expression, process rates or causal interactions, so interpretation requires caution (Pérez-Losada et al., 2022). China’s large wood-decaying mushroom industry generates substantial quantities of lignocellulosic SMS. *Auricularia heimuer* and *Flammulina velutipes* were selected because they are widely cultivated, high-output species representing wood-shaving-based and corn-cob-based production systems, respectively. Here, we compared conventional composting and *Eisenia fetida*-mediated composting of seven mixtures of black fungus SMS (AhSMS) and golden needle mushroom SMS (FfSMS) over 0, 30 and 60 d. We combined physicochemical measurements, 16S rRNA gene sequencing, PICRUSt2-based functional prediction and microbial network analysis to assess bacterial succession, nitrogen mineralization and candidate taxa associated with nitrogen-cycling outcomes.

## 2 Materials and methods

### 2.1 Experimental site

The experiment was carried out in the greenhouse of Jilin Provincial Vegetable and Flower Research Institute, Changchun, Jilin Province, China (43°53′24″N, 125°39′56″E) in May 2025. Both conventional and earthworm-mediated treatments were set up in the same greenhouse to allow comparing composting mode, substrate composition and sampling time under a same experimental circumstance.

### 2.2 Experimental materials

The earthworms (*E. fetida*) were collected from Donggou Farm in Tumen City, Yanbian Prefecture, Jilin Province. Before addition, worms were acclimated to the experimental SMS for 7 d under greenhouse conditions. Each 5-kg container in Group E received 100 worms, corresponding to 100 g fresh mass; worms were approximately mixed juvenile and clitellate adults. The substrate gradient was made using two types of SMS. Black fungus SMS (AhSMS), derived mainly from wood shavings, was supplied by Fuzhidao (Jilin) Biotechnology Co., Ltd. Golden needle mushroom SMS (FfSMS), derived mainly from corn cobs, was supplied by Changchun Gaorong Biotechnology Co., Ltd. AhSMS and FfSMS were combined to represent contrasting wood-shaving-based and corn-cob-based residues and to evaluate substrate-dependent composting responses across a controlled mixing gradient. Both materials were air-dried to about 40% moisture content before setting up the treatments and subsequently kept at 4 °C so as to maintain their microbial and chemical properties. The same pretreatment was performed prior to being mixed in the proportions mentioned below.

### 2.3 Experimental design and sampling

#### 2.3.1 Experimental design

The experiment was a two factor design including the composting mode and substrate composition. A comparison was made between conventional composting (without earthworms) (Group C) and earthworm mediated composting with *E. fetida* (Group E). For each mode, a gradient of seven AhSMS–FfSMS mixtures was made ranging from 100% FfSMS to 100% AhSMS (Table 1). The T1–T7 treatment codes indicate the different substrate ratios and the suffix E or C the different composting modes. The 14 combinations of treatments were replicated three times providing 42 experimental containers.

**Table 1:** SMS mixing ratios.

Test area number	Test group number	Compost matrix
Earthworm (E)	T1(E)	*Ff*SMS 100%
	T2(E)	*Ff*SMS 75% + *Ah*SMS 25%
	T3(E)	*Ff*SMS 67% + *Ah*SMS 33%
	T4(E)	*Ff*SMS 50% + *Ah*SMS 50%
	T5(E)	*Ff*SMS 33% + *Ah*SMS 67%
	T6(E)	*Ff*SMS 25% + *Ah*SMS 75%
	T7(E)	*Ah*SMS 100%
Control (C)	T1(C)	*Ff*SMS 100%
	T2(C)	*Ff*SMS 75% + *Ah*SMS 25%
	T3(C)	*Ff*SMS 67% + *Ah*SMS 33%
	T4(C)	*Ff*SMS 50% + *Ah*SMS 50%
	T5(C)	*Ff*SMS 33% + *Ah*SMS 67%
	T6(C)	*Ff*SMS 25% + *Ah*SMS 75%
	T7(C)	*Ah*SMS 100%

The composting was carried out in plastic containers 40 × 31 × 35 cm in size with perforations. The containers were covered with wire mesh to allow ventilation but control insect entry. The experimental unit was a container containing 5 kg of the respective SMS mixture. The addition of earthworms was done in Group E, while the Group C containers were kept without earthworms. Moisture was maintained at approximately 60%–70% (w/w) by weekly gravimetric checks and addition of deionized water as needed. Greenhouse temperature ranged from 20 °C to 28 °C. Materials were gently mixed every 10 d. Temperature was recorded only as a greenhouse-level range, and moisture was checked weekly rather than monitored continuously within each container. Substrate pH and earthworm biomass, survival and feeding activity were not monitored during composting.

#### 2.3.2 Sampling methods

Sample testing was done at the beginning of the experiment, at 30 d, and at 60 d of composting. Material in each replicate container was homogenized at each time point to minimize microscale heterogeneity. A composite sample of 80 g was taken and split for subsequent analyses downstream. A 50 g subsample was air-dried for physicochemical measurements and a 30 g subsample was placed in a −80 °C freezer for DNA extraction and 16S rRNA gene sequencing. This allowed the evaluation of both the physicochemical changes and the bacterial community patterns from the same homogenized material.

### 2.4 Physicochemical and molecular analyses

#### 2.4.1 Physicochemical analysis

Bulk carbon and nitrogen pools, mineral nitrogen availability and extracellular enzyme activity were used to describe compost transformation. The eight measured variables were total carbon (TC), total nitrogen (TN), organic matter (OM), ammonium nitrogen (NH_4_^+_^N), nitrate nitrogen (NO_3_^−_^N), alkali-hydrolyzable nitrogen (AN), urease activity (UE) and alkaline protease activity (AP). An additional measure of substrate stoichiometry was the C/N ratio, which was determined from TC and TN.

TC was analysed by a Unicube elemental analyzer (Elementar, Germany) (Nelson and Sommers, 1996). The OM was determined by oxidation with potassium dichromate (Walkley & Black Walkley and Black, 1934) and TN was determined by the Kjeldahl method (Bremner, 1996). Extraction and quantification of NH_4_^+_^N and NO_3_^−_^N were done by standard colorimetric procedures, while AN was used to characterize the readily mineralizable nitrogen pool. Soil enzymes as indicators of UE and AP were determined by standard soil enzyme procedures (Kandeler and Gerber, 1988; Tabatabai and Bremner, 1972). The total variable set was applied to separate the total nutrient pool changes from the inorganic N accumulation and enzyme mediated N transformation changes.

#### 2.4.2 16S rRNA gene sequencing

Total genomic DNA was extracted from 180 to 220 mg of frozen compost by using the TIANamp Stool DNA Kit (Tiangen, Beijing). The V4 region of the 16S rRNA gene of bacteria was amplified using the primer pair 515F (GTGCCAGCMGCCGCGGTAA) and 806R (GGACTACHVGGGTWTCTAAT). Novogene (Beijing, China) used Illumina NovaSeq 6000 platform paired-end 250-bp to perform amplicon library sequencing. Raw reads and sample metadata have been deposited in NCBI Sequence Read Archive (BioProject PRJNA1405922; https://www.ncbi.nlm.nih.gov/bioproject/PRJNA1405922).

Amplicon sequence variants (ASVs) were resolved using Divisive Amplicon Denoising Algorithm 2 (DADA2) in Quantitative Insights Into Microbial Ecology 2 (QIIME2) with quality-filtered raw reads. Taxonomic identification was done based on the SILVA database. The output tables of ASV abundance and taxonomy were used to estimate the alpha-diversity, perform Bray–Curtis dissimilarity analysis, taxonomic profiling, functional prediction with PICRUSt2, and microbial network construction. This resulted in a workflow where the ASV approach maintained the resolution of the sequences (no reads were clustered into operational taxonomic units).

### 2.5 Statistical analysis and functional inference

The ASV abundance matrix was used to infer the microbial functional profiles with PICRUSt2 (v 2.3.0). For comparison downstream, enzyme commission (EC) categories were identified that are associated with nitrogen cycling and were extracted. As PICRUSt2 estimates functional potential based upon taxonomic marker-gene data, the resulting abundance of ECs was considered as inferred pathway potential, rather than actual measures of gene abundance, transcription, or process rates.

**Figure 1:**
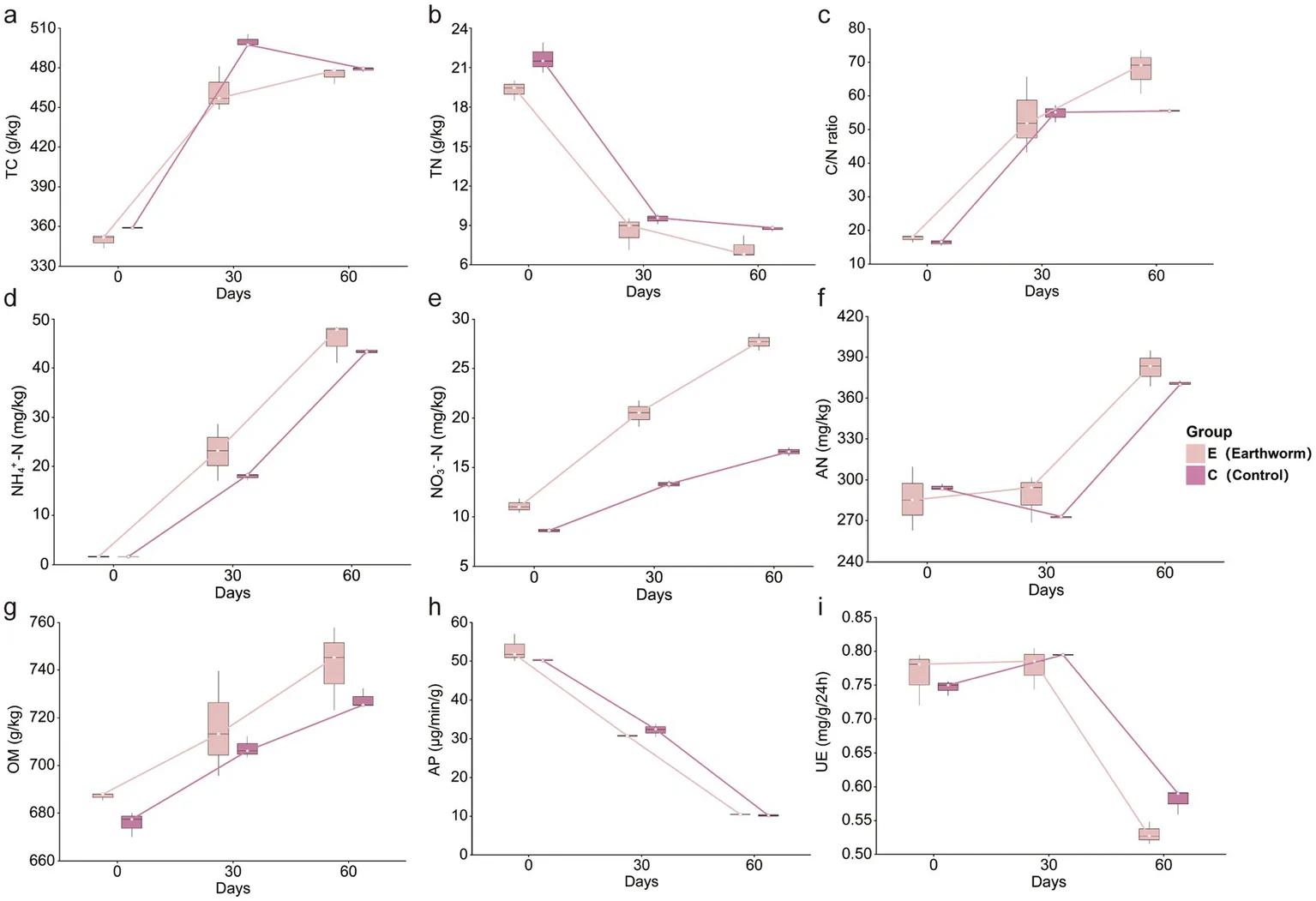
Temporal trajectories of physicochemical variables during conventional and earthworm composting. Panels show total carbon **(a)**, total nitrogen **(b)**, carbon-to-nitrogen ratio **(c)**, ammonium nitrogen **(d)**, nitrate nitrogen **(e)**, alkali-hydrolyzable nitrogen **(f)**, organic matter **(g)**, alkaline protease activity **(h)** and urease activity **(i)**. “Earthworm” denotes the earthworm composting treatment, and “Control” represents conventional composting. At each mode-by-day combination, the boxplot summarizes the seven substrate-ratio means (T1–T7); each ratio mean was calculated from three independent containers, so each box represents seven ratio means derived from 21 underlying container observations. The center line denotes the median, the box denotes the interquartile range, the whiskers extend to the most extreme values within 1.5 × the interquartile range, and the white point denotes the mean. Lines connect the mode-level means across sampling times. Values for individual treatment combinations are mean ± *SD* (*n* = 3) in Supplementary Table S1; exact omnibus *p* values and sample-size definitions are provided in Supplementary Table S4.

**Figure 2:**
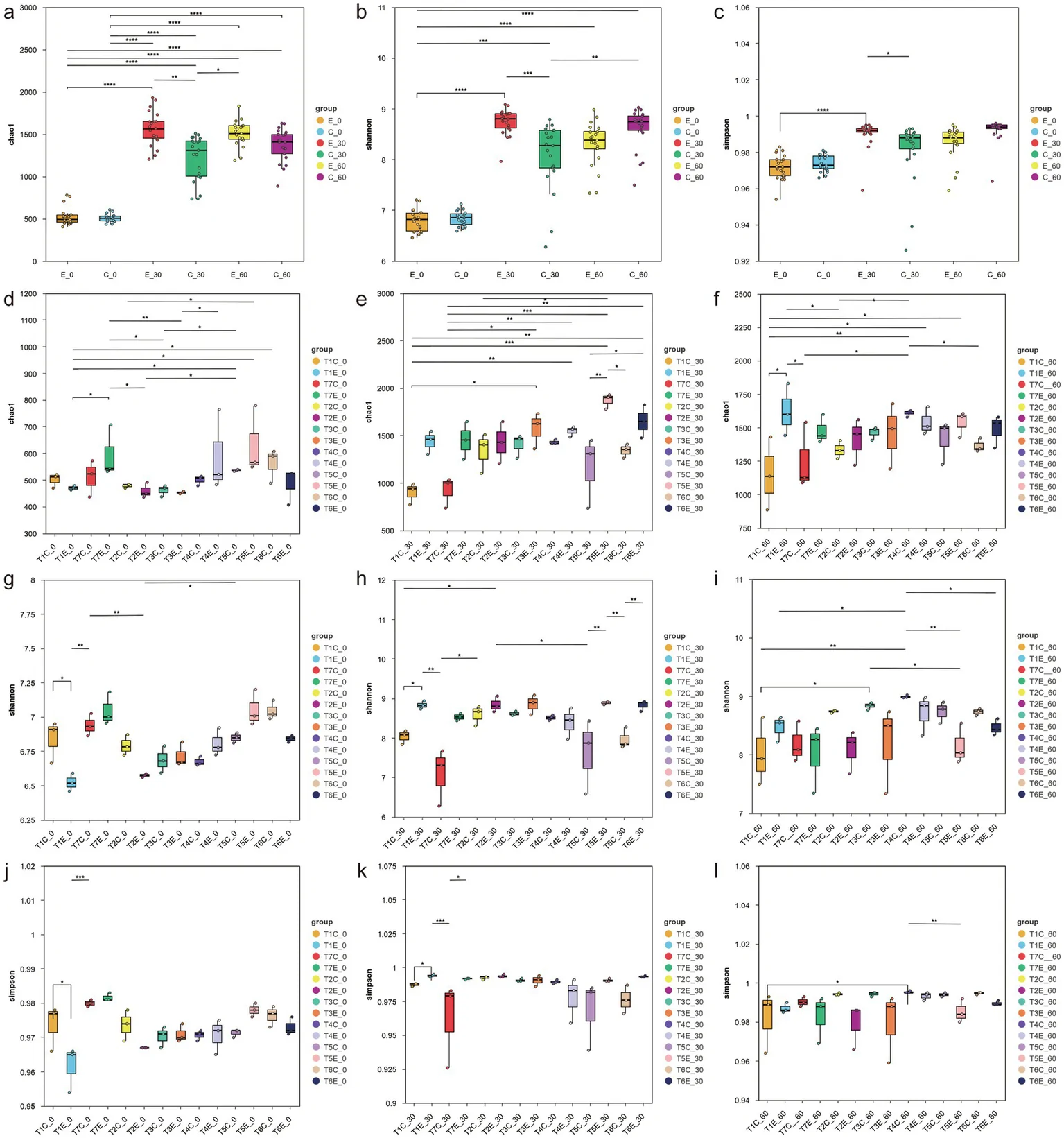
Bacterial alpha diversity across composting modes, substrate mixtures and time. Panels **(a–c)** compare Chao1, Shannon and Simpson indices between conventional and earthworm composting at 0, 30 and 60 d; each mode-by-day box contains 21 container observations pooled across T1–T7. Panels **(d–f)**, **(g–i)**, and **(j–l)** show Chao1, Shannon and Simpson distributions, respectively, across T1–T7 at each sampling time; each mode-by-ratio-by-day box contains three independent container observations. The center line denotes the median, the box denotes the interquartile range, the whiskers extend to the most extreme values within 1.5 × the interquartile range, and points represent individual observations. Labels combine substrate code, composting mode and day. Mean ± SD summaries for Chao1 and Shannon are provided in Supplementary Table S2.

The 42 containers (14 treatment combinations × 3 independent containers) were the experimental units. The same containers were sampled at 0, 30 and 60 d; observations across time were therefore longitudinal. For physicochemical variables, one-way ANOVA followed by Duncan’s multiple-range test (SPSS 22.0) was used for separate within-time comparisons among the 14 combinations (C/E × T1–T7), with *n* = 3 containers per combination. Values in Supplementary Table S1 are reported as mean ± SD. In Figure 1, each mode-by-day box summarizes the seven substrate-ratio means (T1–T7), with each ratio mean calculated from three containers; thus, each box represents seven ratio means derived from 21 underlying container observations. In Figures 2a–c, each mode-by-day box contains 21 container observations pooled across T1–T7; in Figures 2d–l, each mode-by-ratio-by-day box contains three container observations. The pooled boxplots were used as descriptive summaries rather than as independent inferential tests of composting mode. Supplementary Table S1 provides Duncan significance letters for the 14 within-time combinations, and Supplementary Table S4 provides sample sizes, the SD definition, the statistical test and exact omnibus *p* values by variable and day. The 22 nitrogen-related EC categories were selected from Supplementary Table S3 by mapping annotations to nitrogen fixation, ammonification, nitrification, denitrification, nitrate/nitrite reduction, urea hydrolysis and glutamate/ammonia assimilation; the complete list is provided in Supplementary Table S3. Candidate genera were selected descriptively from taxa with marked temporal changes in genus-level abundance or from network-central taxa. This exploratory selection did not constitute differential-abundance testing. Genus–EC correlations are associations within the same 16S-derived dataset, not independent functional validation.

**Figure 3:**
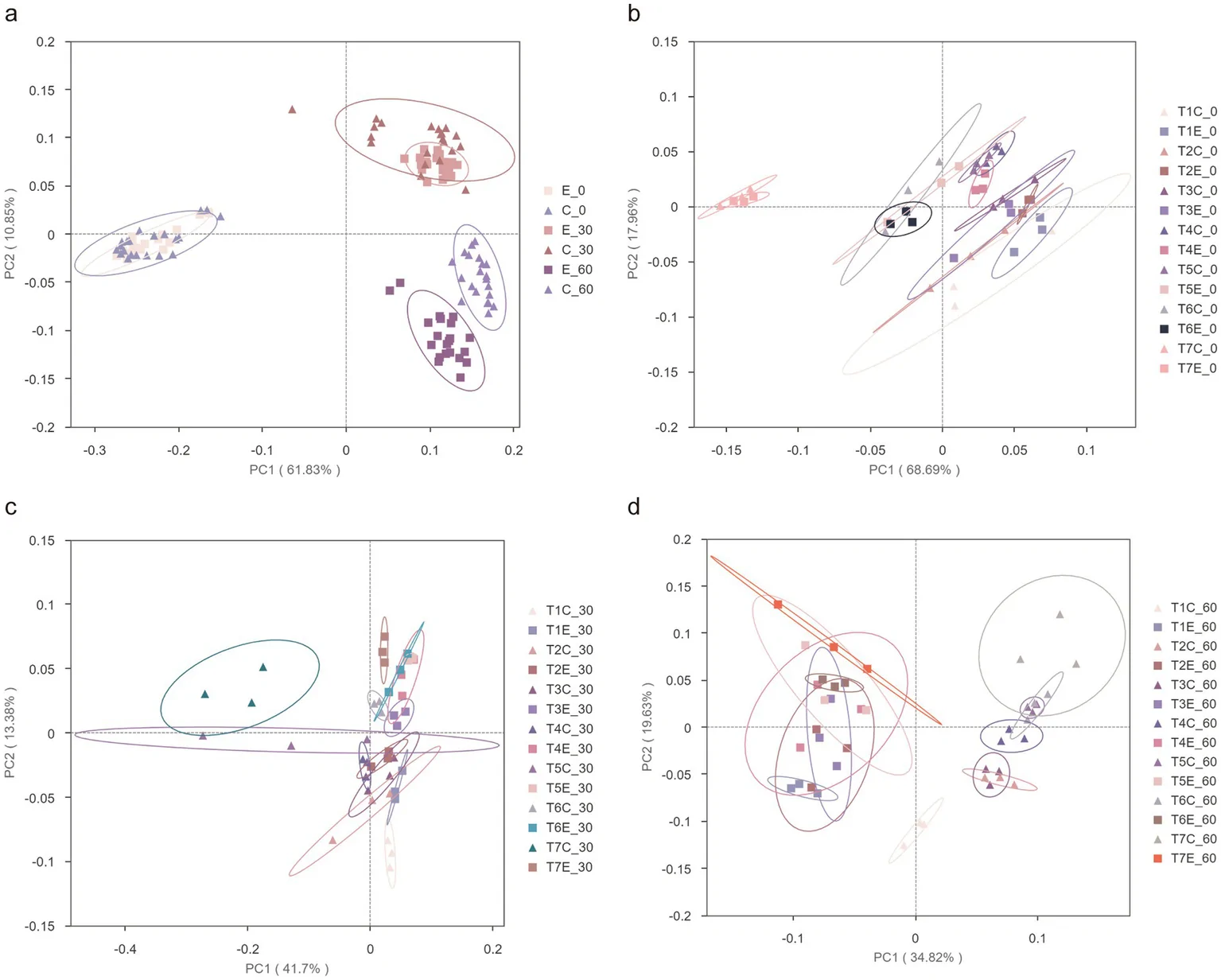
Temporal and treatment-associated beta-diversity patterns revealed by Bray–Curtis principal coordinate analysis (PCoA). Panel **(a)** groups samples by composting duration (0, 30 and 60 d). Panels **(b–d)** compare conventional and earthworm composting at days 0, 30 and 60, respectively. PERMANOVA identified a significant day effect (*R*^2^ = 0.4915, *p* < 0.001). Composting mode was not significant at day 0 (*R^2^* = 0.0052, *p* = 0.146), but was significant at day 30 (*R^2^* = 0.2183, *p* < 0.001) and day 60 (*R*^2^ = 0.3112, *p* < 0.001). Multivariate dispersion between Groups C and E was homogeneous within each day (PERMDISP, *p* > 0.05).

Beta diversity was analysed using Bray–Curtis dissimilarities calculated from the even-depth ASV Table (35,497 reads per sample). PERMANOVA was performed with adonis2 in vegan v2.7–5 under R v4.6.1, using 9,999 permutations. The global model included sampling day, composting mode, substrate mixture and all interactions. Repeated sampling was accommodated using a split-plot permutation design. Container (substrate mixture × composting mode × replicate) was treated as the whole plot, and sampling day was permuted within each container. Separate within-day models corresponding to Figures 3b–d tested substrate mixture, composting mode and their interaction. Homogeneity of multivariate dispersion was assessed by PERMDISP, based on distances to group centroids and 9,999 permutations.

**Figure 4:**
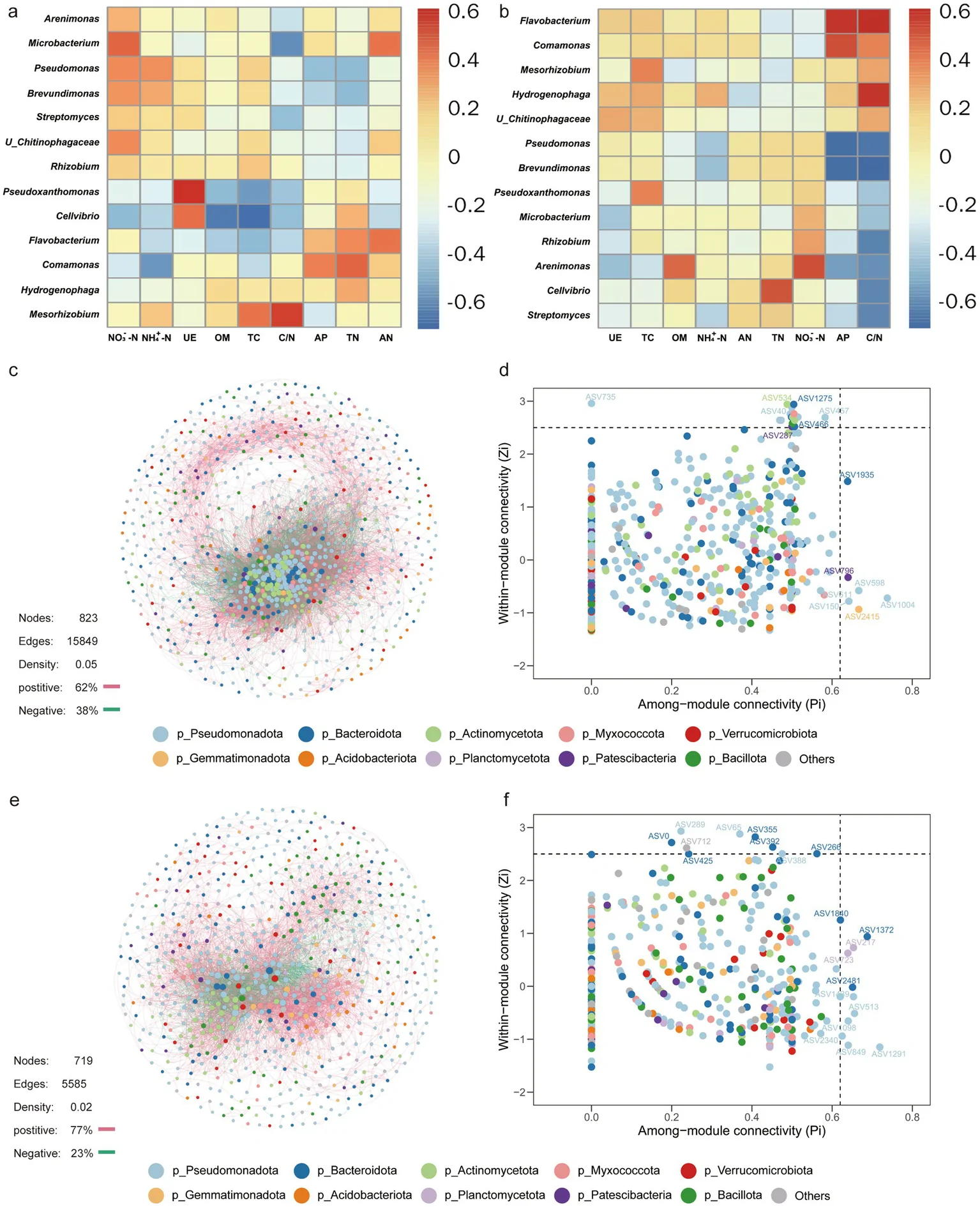
Microbial–environmental correlations and ASV co-occurrence networks on day 60. Panels **(a,b)** show Spearman correlation heatmaps for selected genera and physicochemical variables in conventional and earthworm composting; red and blue indicate positive and negative correlations, respectively, and “U” denotes an unclassified taxon. Panels **(c,e)** show the corresponding ASV networks; node color denotes phylum, node size denotes relative abundance, and red and green edges denote positive and negative associations. Summaries report node number, edge number, density and edge-sign proportions. Panels **(d,f)** show module roles based on Zi and Pi, with dashed boundaries at 2.5 and 0.62.

To link physicochemical and taxonomic patterns directly, Spearman correlations were calculated at day 60 between selected genus abundances and physicochemical variables within each composting mode. The resulting coefficients were visualized as genus–environment heatmaps (Figures 4a,b). Each taxonomic profile corresponded to physicochemical measurements obtained from the same homogenized material. These correlations were interpreted as associations, not as evidence of causal microbial control over nitrogen fluxes.

### 2.6 Network construction and analysis

#### 2.6.1 Co-occurrence network analysis

Bacterial co-occurrence networks were constructed in R 4.5.2 using ASVs with relative abundance greater than 0.01%. Pairwise Spearman correlation coefficients were calculated to represent abundance associations among retained ASVs. Edges were retained when |*r*| > 0.8 and *p* < 0.01, and isolated nodes were removed before calculation of network properties. In the resulting networks, nodes represented ASVs and edges represented significant positive or negative correlations rather than direct biological interactions.

The igraph package was used to calculate node number, edge number and network density. Taxonomic information was mapped to the nodes to compare the organisms contributing to each treatment network. Module roles were evaluated from within-module connectivity (Zi) and among-module connectivity (Pi), using Zi = 2.5 and Pi = 0.62 as the displayed classification boundaries. These metrics were used to distinguish peripheral nodes from nodes with high connectivity within or among network modules.

#### 2.6.2 Genus–function association networks

Associations between nitrogen-cycling bacterial genera and predicted EC categories were evaluated using Pearson correlation analysis. Relationships were retained at |*r*| > 0.9 and *p* < 0.05 and were visualized in Gephi 10.1. Bacterial genera and EC categories were represented as separate node classes, whereas edge color indicated positive or negative associations and node degree summarized the number of retained connections. These interaction networks were used to identify candidate genera linked to multiple nitrogen-related functions, while recognizing that correlation does not establish direct metabolic exchange or causality.

**Figure 5:**
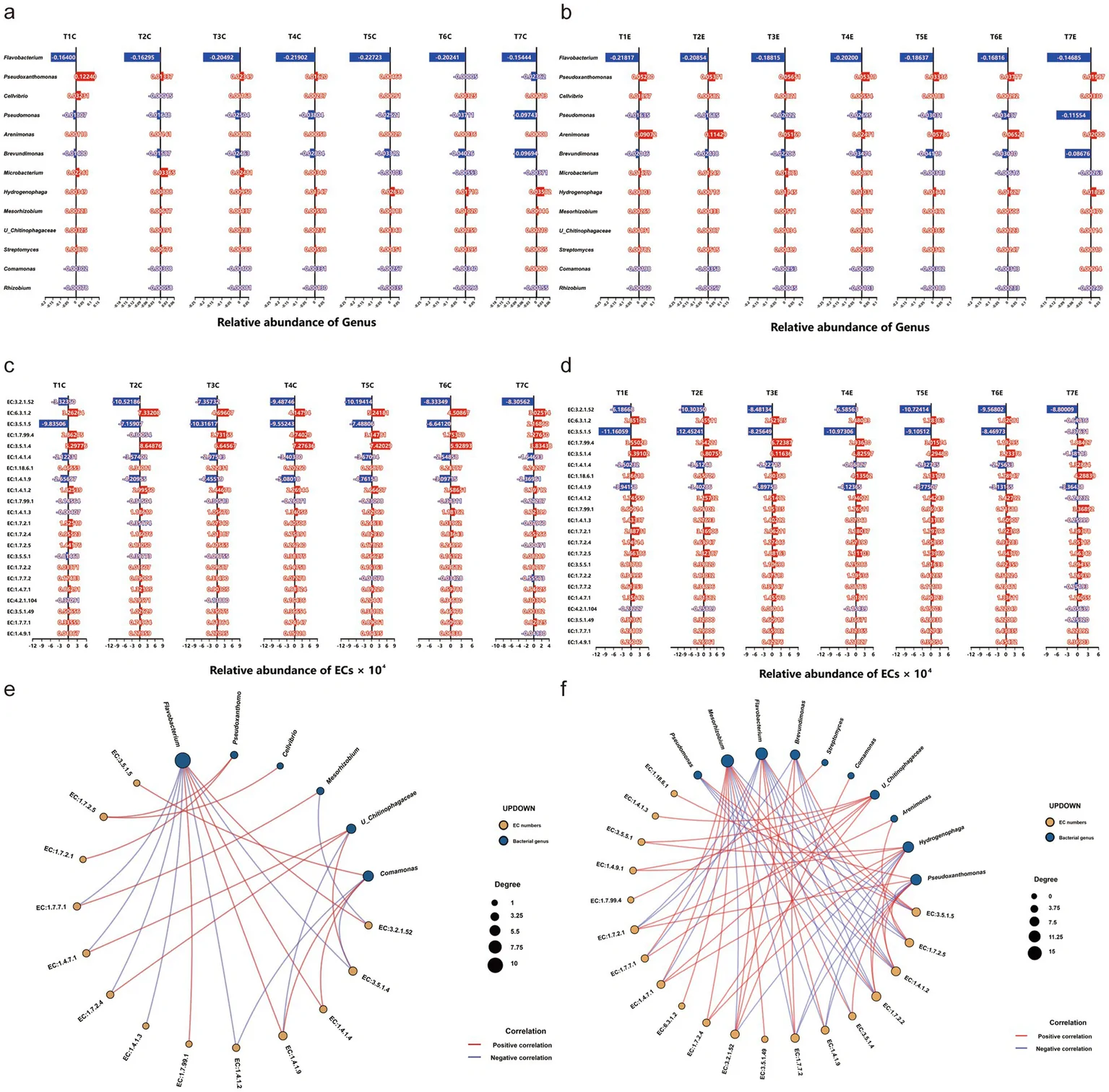
Changes in candidate nitrogen-cycling genera, predicted enzyme commission (EC) categories and genus–EC association networks. Panels **(a,b)** show changes in the relative abundance of selected genera from day 0 to day 60 in conventional and earthworm composting, respectively. Panels **(c,d)** show changes in predicted nitrogen-cycle EC categories (values ×10^4^) in the corresponding treatments. Red and blue values indicate increases and decreases, respectively; “U” denotes an unclassified taxon. Panels **(e,f)** show Pearson correlation networks between genera and predicted EC categories in conventional and earthworm composting, respectively. Edges were retained at |*r*| > 0.9 and *p* < 0.05. Yellow nodes represent EC categories, blue nodes represent bacterial genera, node size indicates degree, and edge color indicates positive (red) or negative (blue) correlation. Networks in panels **(e,f)** were based on day-0-to-day-60 change values across T1–T7 within each composting mode; day-30 observations were excluded. Maximum displayed node degrees were 10 and 15, respectively; these descriptive values were not tested as a between-network difference.

The networks in Figures 5e,f were constructed separately for conventional and earthworm composting from day-0-to-day-60 change values across the seven substrate mixtures (T1–T7); day-30 observations were excluded. Each edge therefore represents a Pearson correlation among substrate-specific endpoint changes within one composting mode, rather than a correlation pooled across all sampling times. Networks were filtered identically using |*r*| > 0.9 and*p*< 0.05, but no permutation or resampling test compared edge counts, degree distributions or global topology. Accordingly, observed network-level differences were treated as descriptive rather than inferential comparisons.

## 3 Results

### 3.1 Physicochemical dynamics during composting

Earthworm composting altered the coordinated trajectories of carbon and nitrogen pools and extracellular enzyme activities during SMS transformation (Figure 1; Supplementary Table S1). Both Group E and Group C changed markedly between day 0 and day 30, followed by smaller changes during the subsequent 30 d. Most variables followed similar temporal directions in the two composting modes, although the magnitude of change diverged over time.

TC increased in both groups, whereas TN declined and the C/N ratio increased. By day 60, TC was slightly higher in Group C (478.93 ± 23.36 g/kg) than in Group E (474.61 ± 20.14 g/kg), a difference of 0.91% (Figure 1a). Conversely, TN was 10% higher in Group E (7.59 ± 2.68 g/kg) than in Group C (6.90 ± 2.18 g/kg) (Figure 1b). The C/N trajectories diverged after day 30, with a lower final ratio in Group E. Using the treatment-level means in Supplementary Table S1, mean NH4^+^-N in Group E increased from 2.34 to 44.17 mg kg^−1^ between day 0 and day 60 (1788%), whereas Group C increased from 2.54 to 39.37 mg kg^−1^ (1450%). Mean NO3^−^-N increased from 10.33 to 26.10 mg kg^−1^ in Group E (153%) and from 9.63 to 16.62 mg kg^−1^ in Group C (73%). These are concentration changes; without dry-mass or nitrogen-balance data they should not be interpreted as greater total nitrogen retention (Figures 1d,e). AN remained near its initial level during the first 30 d before increasing sharply in both groups, with a higher final value in Group E (Figure 1f). OM also increased throughout composting and remained higher in Group E at day 60 (Figure 1g).

Across all mixture–mode combinations, measured initial C/N ratios ranged from 16.38 to 61.38 (Supplementary Table S1). For T3 in Group E, the C/N ratio was 17.64 ± 1.10, 53.54 ± 1.31 and 47.75 ± 1.58 at days 0, 30 and 60, respectively. The corresponding values in Group C were 16.38 ± 0.96, 54.81 ± 2.54 and 55.58 ± 0.28.

The pathways of AP and UE were different from those of inorganic nitrogen. The activity of AP decreased consistently in both groups and reached the same low level by day 60 (Figure 1h). Although little variation in UE was observed in the first 30 d, it dropped drastically thereafter, and more so in Group E (Figure 1i). Thus, the decline in enzyme activities did not parallel the increases in NH_4_^+_^N, NO_3_^−_^N and AN, indicating that endpoint enzyme assays did not track inorganic nitrogen accumulation in a simple one-to-one manner.

### 3.2 Bacterial diversity dynamics

#### 3.2.1 Alpha diversity

Bacterial alpha-diversity values generally increased during SMS composting, particularly during the first 30 d (Figures 2a–c). On day 0, the distributions of Chao1, Shannon and Simpson were similar for Group E and Group C. Chao1 and Shannon values increased in the reported summaries at days 30 and 60, whereas Simpson is shown in Figure 2; these patterns suggest that the initial communities became more complex and even during composting.

The estimated richness differed among groups. Chao1 and Shannon values in Supplementary Table S2 are reported as mean ± SD (*n* = 3 containers per treatment combination) for each composting mode, substrate ratio and sampling time. Simpson values are shown in Figure 2 but are not included in the Supplementary Table S2 summary.

Shannon diversity generally exhibited a trend similar to Chao1, though it peaked in treatment T4 of Group C and treatment T3 of Group E (Figures 2g–i). Some differences were observed between Simpson values, although the majority of treatments had values higher than those of the initial day for both days 30 and 60 (Figures 2j–l). In combination, these findings indicate that the composting process led to higher bacterial richness and diversity, with varying relative abundance of dominant groups depending on the substrates.

#### 3.2.2 Beta diversity

PCoA revealed a strong temporal shift in bacterial community composition (Figure 3a). Across all samples, the first two principal coordinates explained 61.83% and 10.85% of the variation, respectively. Day-0 samples occupied a distinct ordination space, whereas day-30 and day-60 samples shifted along PC1 and separated further along PC2. Repeated-measures PERMANOVA identified a significant effect of sampling day (*R^2^* = 0.4915, pseudo-*F* = 59.44, *p* < 0.001). Multivariate dispersion also differed among days (PERMDISP*F* = 56.92, *p* < 0.001). The temporal PERMANOVA therefore captured both centroid displacement and variation in within-day dispersion.

At day 0, PC1 and PC2 explained 68.69 and 17.96% of the variation, respectively (Figure 3b). After accounting for substrate mixture, community composition did not differ significantly between Groups E and C (*R^2^* = 0.0052, pseudo-*F* = 2.01, *p* = 0.146). Multivariate dispersion was also comparable between modes (PERMDISP*P* = 0.872). However, the substrate mixture × composting mode interaction was significant (*R^2^* = 0.0379, pseudo-*F* = 2.43, *p* = 0.026). Thus, initial mode-dependent differences varied among substrate mixtures despite the absence of an overall mode effect.

At day 30, PC1 and PC2 explained 41.70 and 13.38% of the variation, respectively (Figure 3c). After accounting for substrate mixture, composting mode explained 21.83% of the Bray–Curtis variation (pseudo-*F* = 27.76, *p* < 0.001). By day 60, the first two coordinates explained 34.82 and 19.63% of the variation, respectively (Figure 3d). The mode effect increased to 31.12% at this time point (pseudo-*F* = 33.72, *p* < 0.001). Dispersion did not differ significantly between Groups E and C at day 30 (PERMDISP *p* = 0.053) or day 60 (*p* = 0.815). These results support differences in group centroids rather than unequal within-group dispersion. Substrate mixture × mode interactions were significant at both time points (*p* < 0.001), indicating that effect magnitude depended on substrate mixture. The global model also identified a day × mode interaction (*R^2^* = 0.0673, pseudo-*F* = 25.89, *p* < 0.001). Community composition therefore changed markedly over time, while separation between composting modes emerged by day 30 and increased by day 60.

### 3.3 Bacterial community composition dynamics

**Figure 6:**
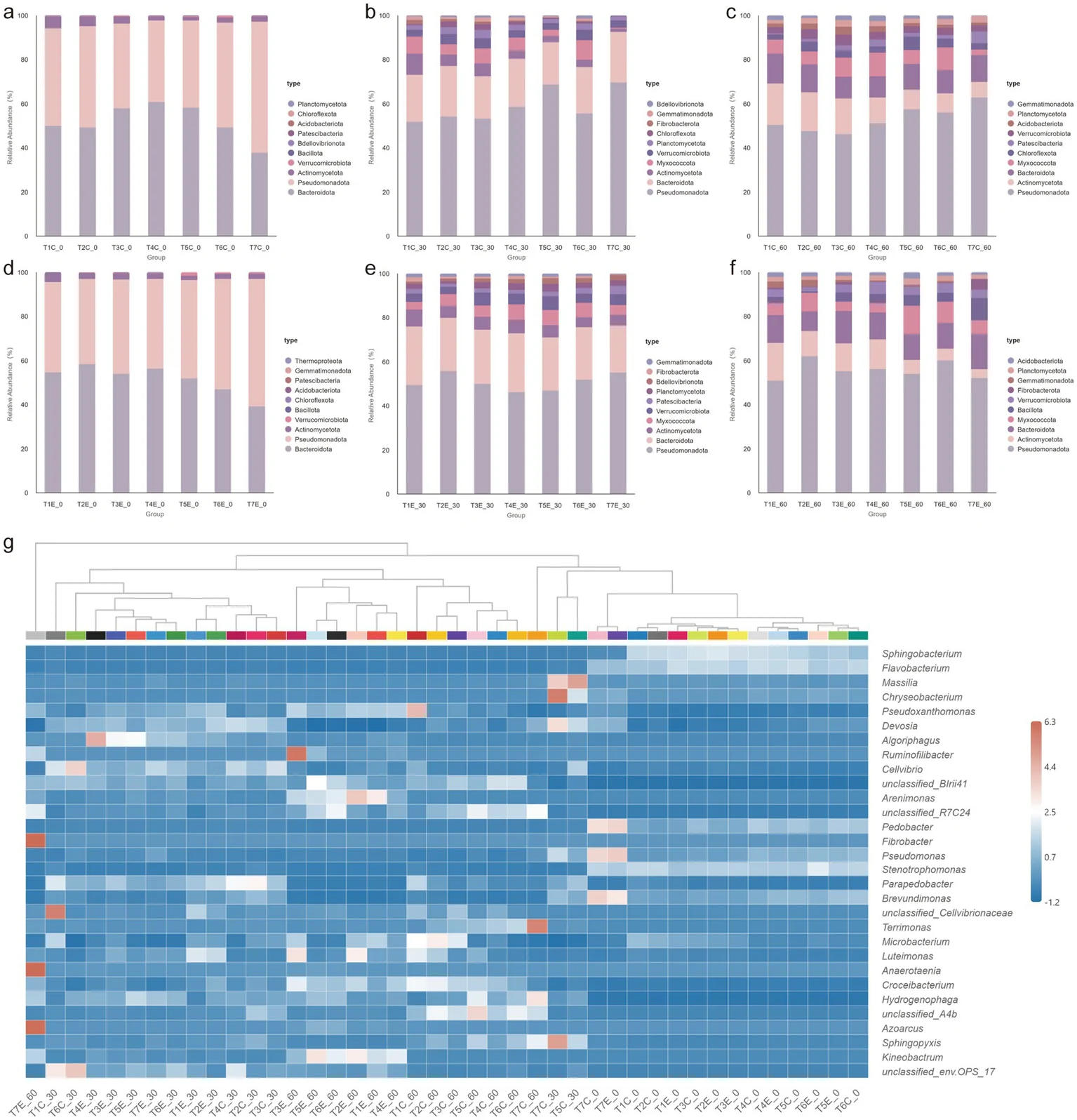
Temporal succession and taxonomic composition of bacterial communities during composting. Panels **(a–c)** show stacked bar charts of the 10 most abundant phyla in conventional composting at 0, 30 and 60 d, respectively. Panels **(d–f)** show the corresponding phylum-level compositions in earthworm composting. Suffixes “0d,” “30d” and “60d” denote composting duration. Panel **(g)** shows descriptive hierarchical clustering of the 30 most abundant genera; color intensity represents standardized relative abundance.

Phylum-level profiles showed a consistent community transition from an initial community dominated by Bacteroidota to a later community dominated by fewer phyla, including higher abundance of Pseudomonadota and Actinomycetota and a few less abundant phyla (Figures 6a–f). Bacteroidota (51.78%), Pseudomonadota (44.57%) and Actinomycetota (3.10%) were the predominant groups at day 0 in Group C. Pseudomonadota rose to 56.63% on day 30 while Bacteroidota decreased to 20.52%. However, other groups, such as Myxococcota (5.14%), Actinomycetota (4.31%) and Verrucomicrobiota (3.72%) also increased in relative abundance. The abundance of Pseudomonadota was still high on day 60, whereas those of Bacteroidota were still decreasing.

The overall successional pattern of group E resembled group C, with the exception of the decrease in Bacteroidota in the late stages. Bacteroidota, Pseudomonadota and Actinomycetota represented 51.51%, 45.12%, and 2.72% of the community, respectively, at day 0. Pseudomonadota increased to 48.16% while Bacteroidota decreased to 23.17% by day 30. The proportions of Pseudomonadota (53.37%), Bacteroidota (12.00%) and Actinomycetota (9.61%) changed at day 60 (Figures 6d–f). Therefore, earthworm composting led to a decrease in the proportion of Bacteroidota and an increase in the proportion of Actinomycetota in the later stages of composting compared with Group C.

The phylum-level profiles varied among the seven substrate mixtures at each sampling date, although most mixtures followed similar temporal trajectories. At day 0, Bacteroidota and Pseudomonadota accounted for nearly all sequences, whereas a broader range of phyla was observed at days 30 and 60. Genus-level profiles further resolved this succession (Figure 6g). The heatmap is descriptive and was not used for statistical differential-abundance testing or direct inference of taxon-specific functions.

### 3.4 Predicted nitrogen-cycle functions and genus–function associations

In general, predicted nitrogen-cycling potential differed across the composting process, but the direction and magnitude of individual EC-category changes were not uniform (Supplementary Table S3; Figure 5). The 22 nitrogen-related EC categories were selected by the mapping criteria described in Section 2.5, and their complete names and EC numbers are listed in Supplementary Table S3. The predicted shifts spanned several nitrogen-transformation processes rather than a single pathway. These values represent PICRUSt2-inferred potential and should not be interpreted as direct measurements of genes, transcripts or process rates.

In Group C, eight of 13 selected genera (61.5%) were found to increase from day 0 to day 60, such as *Pseudoxanthomonas* (+251.91%), *Microbacterium* (+67.04%) and *Hydrogenophaga* (+4238.52%). *Flavobacterium*, on the other hand, declined by 98.19%. Such opposing responses reflect taxonomic turnover and selective enrichment; not uniform enrichment of all candidate nitrogen-cycling taxa.

Group E was heterogeneous across the seven substrate mixtures (Figures 5b,d). Multiple nitrogen-related EC categories changed alongside candidate genera, although directions and magnitudes varied among substrate ratios. The networks in Figures 5e,f summarized day-0-to-day-60 change correlations across T1–T7 separately for each composting mode; day-30 observations were not included. Under the common filtering threshold, the maximum displayed node degree was 15 in the earthworm network and 10 in the conventional network. No permutation or resampling test compared edge counts, degree distributions or overall topology between networks. We therefore interpret Figures 5e,f as descriptive evidence of different retained association patterns, not as evidence that the earthworm network was more complex. Several genera connected to multiple EC categories, supporting an overlapping rather than one-genus–one-function structure.

These results represent functional predictions derived from 16S rRNA gene profiles rather than direct measurements of genes or transcripts. Nevertheless, the concurrent increases in inorganic nitrogen, selected bacterial genera and predicted EC categories show that bacterial succession accompanied shifts in nitrogen-cycling potential. Correlations with NH_4_^+_^N and NO_3_^−_^N further identified candidate taxa and pathways for metagenomic, metatranscriptomic or activity-based validation.

### 3.5 Microbial network structure and environmental associations

By day 60, microbial–environmental correlation patterns differed between conventional and earthworm composting (Figures 4a,b). In Group C, NO_3_^−_^N was positively correlated with *Arenimonas*, *Microbacterium*, *Pseudomonas*, *Brevundimonas* and U_Chitinophagaceae. NH_4_^+_^N was positively correlated with *Pseudomonas*, *Brevundimonas* and *Mesorhizobium*. In Group E, the correlation structure was more selective: NO_3_^−_^N was positively associated with *Arenimonas*, *Rhizobium* and *Microbacterium*, whereas NH_4_^+_^N was associated with *Hydrogenophaga*. TN was positively associated with *Cellvibrio*, and TC was positively associated with *Mesorhizobium* and *Pseudoxanthomonas*. Several genus–environment correlations also changed in direction or magnitude between treatments.

These genus–environment correlations directly linked the day-60 inorganic-N patterns in Figure 1 to the taxonomic shifts resolved in Figure 6. The shared NO₃^−^-N associations and treatment-specific NH₄^+^-N associations show that inorganic-N endpoints were embedded within different genus-level configurations. Thus, higher inorganic-N concentrations did not merely occur alongside community change; they covaried with specific components of the reassembled communities.

The ASV co-occurrence networks differed in size, density and edge composition (Figures 4c,e). The conventional network contained 823 nodes and 15,849 edges, with a density of 0.05; 62% of edges were positive and 38% were negative. The earthworm network contained 719 nodes and 5,585 edges, with a density of 0.02; 77% of edges were positive and 23% were negative. Thus, earthworm composting was associated with a smaller, less dense network but a higher proportion of positive associations.

Module-role analysis identified a limited number of highly connected ASVs in each network (Figures 4d,f). In Group C, *Microbacterium*, *Cupriavidus* and *Devosia* were module hubs, whereas *Sphingopyxis* acted as an intermodule connector. Group E was characterized by the central network positions of *Flavobacterium*, *Cellvibrio* and *Rhizobium*. These shifts in the central taxa, along with changes in the structure of nodes and links, provide a hint of the potential for reorganization of the roles within the bacterial community in response to treatment. Together, the smaller and less dense ASV network, higher proportion of positive edges and different central taxa indicate treatment-associated network reorganization rather than greater connectivity.

## 4 Discussion

### 4.1 Earthworm-associated community reassembly and higher inorganic-N concentrations

The principal observation was that the *E. fetida* composting was associated with higher inorganic-N concentrations and a distinct pattern of bacterial community reassembly. By day 60, Group E showed higher NH_4_^+_^N and NO_3_^−_^N, respectively, together with higher TN than the conventional treatment. This early increase in diversity and the tendency towards different configurations in the later stages suggests a dual effect of earthworm treatment on community expansion and selection of alternative assemblages. This interpretation aligns with the ability of earthworms to fragment and mix, alter substrate accessibility and microscale habitats for microorganisms, which can affect decomposition (Gong et al., 2019; Buivydaitė et al., 2023). Further evidence of vermicomposting indicates that bacterial communities vary across earthworm gut compartments, suggesting that gut passage can also help shape microbial selection, in addition to just accompanying it (Zhao et al., 2023). The inorganic-N pools should nevertheless be regarded as the net result of the depolymerization of organic-N, the ammonification, the microbial immobilization, the nitrification and a possible gaseous loss. The coupled increase in NH_4_^+_^N, NO_3_^−_^N and alkali-hydrolysable N was consistent with higher measured plant-available N pools, but it does not resolve the relative contribution of each process.

The temporal displacement in Figure 3 captures the temporal component of community reassembly, whereas Figure 4 identifies day-60 genera that covaried with the nitrogen pools in Figure 1. The shared NO₃^−^-N associations and treatment-specific NH₄^+^-N associations indicate that inorganic-N accumulation occurred within distinct taxonomic configurations, rather than alongside a uniform community response. This correspondence strengthens the association between community reassembly and nitrogen availability, but it does not establish microbial causation or enhanced mineralization rates.

A key limitation is that the experiment did not resolve direct earthworm effects from indirect effects mediated through altered environmental conditions. Container-specific temperature, pH and moisture trajectories were unavailable, and earthworm biomass, survival and feeding activity were not measured over time. Consequently, the observed microbial and inorganic-N changes should be interpreted as earthworm-associated rather than as effects caused solely by direct feeding or gut passage. Earthworms may also have acted indirectly by modifying aeration, substrate structure, moisture distribution, pH or temperature. Future experiments should combine continuous environmental monitoring with repeated earthworm-performance measurements and mediation or factorial analyses to separate these pathways.

### 4.2 Successional specialization in the nitrogen-cycling microbiome

The main axis of community change was time, and earthworm treatment and substrate ratio increasingly differentiated communities at later time. The relative abundance of Pseudomonadota and Actinomycetota was higher in the latter assemblage, which gradually replaced the early assemblage containing a higher proportion of Bacteroidota, and the change was more pronounced in Group E on day 60. Ecologically this trend is consistent with a shift from fast use of readily available organic matter to the use of more chemically heterogeneous residues. It also offers a microbial perspective on the lagged response of inorganic N increases, most evident after day 30. A similar link between community composition, functional-gene profiles and nitrogen transformation has been found in managed composting systems (Wang et al., 2022). Taxonomic changes, however, cannot pinpoint the organisms behind individual nitrogen transformations, since there are functional traits within phyla and genera. Hence, the succession can be considered as community level reorganization of nitrogen pools and not as taxonomic evidence of a particular biochemical pathway.

Genus-level evidence distinguishes plausible nitrogen-cycle functions from unsupported functional assignments. Some *Pseudoxanthomonas* strains reduce nitrate or nitrite and, in some cases, produce N₂O, suggesting strain-specific roles in nitrate reduction or denitrification (Finkmann et al., 2000; Mohapatra et al., 2018). However, these observations do not establish organic-N mineralization or ammonification as genus-wide functions. In contrast, characterized *Arenimonas* strains are generally non-nitrate-reducing (Chen et al., 2015). C*roceibacterium*is represented mainly by taxonomic and environmental isolation studies, whereas direct nitrogen-transforming functions remain unresolved for both *Croceibacterium* and *Kineobacterium* (Liu et al., 2019). Their enrichment may therefore reflect substrate turnover or shared environmental responses rather than direct nitrogen mineralization. Without taxon-resolved functional genes or process-rate measurements, these genera cannot be assigned as drivers of NH₄^+^-N or NO₃^−^-N accumulation.

Substrate composition modulated this succession, and T3 comprised 67% FfSMS and 33% lignin-rich AhSMS. However, its C/N trajectory did not provide evidence of superior performance relative to the other mixtures (Section 3.1; Supplementary Table S1). T3 should therefore be regarded as a candidate blend for further investigation, rather than as a rational or optimal formulation.

### 4.3 Candidate hub taxa and limits of network inference

The network analyses identify taxa at the hub of the community, including *Mesorhizobium* and *Flavobacterium* species, which are candidates for the hub at the junction between community structure and predicted nitrogen-assimilation potential. They have positive associations with glutamate synthase and glutamine synthetase categories that could be interpreted as involvement in ammonia assimilation, a pivot-point in the microbial nitrogen-cycling network (Kuypers et al., 2018). More generally the earthworm treatment altered central ASVs, decreased total network size, decreased total network density and increased the percentage of positive edges. This combination indicates that the community was not simply more connected as a result of earthworm composting. Rather it took another course of co-occurrence that may have been related to similar reactions to the substrate or to different ecological niches. Positive co-occurrence edges may stem from some common environmental choices, and hub status should be used as a prioritization criteria for experimental tests. *Flavobacterium* can be a potential contributor to the processing of organic matter, which may indirectly contribute to the nitrogen transformation downstream by providing lower molecular weight substrates, but it is still a testable hypothesis in SMS composting.

More specifically, M*esorhizobium*is best known for host-dependent symbiotic N₂ fixation, but its occurrence in SMS does not establish active fixation or NH₄^+^-N release (Laranjo et al., 2014). Associations with glutamine synthetase and glutamate synthase are equally consistent with ammonium assimilation, which consumes rather than produces NH₄^+^-N. *Flavobacterium* includes protein- and polysaccharide-degrading species that could indirectly facilitate ammonification by releasing low-molecular-weight organic substrates (McBride et al., 2009). Some *Flavobacterium* species also reduce nitrate and nitrite under anoxic conditions, although this trait is not genus-wide (Horn et al., 2005). Thus, network centrality supports indirect links through substrate processing, nitrogen assimilation or community interactions, rather than direct NH₄^+^-N or NO₃^−^-N production or consumption.

### 4.4 Reconciling enzyme activity and predicted functional potential

One of the interesting aspects of the results was that the extracellular enzyme activities decreased while the predicted nitrogen-cycling potential increased. In Group C, 77% of the selected nitrogen-cycle EC categories had increased from day 0 to day 60 while urease activity had decreased by 31% and alkaline-protease activity by 80%. These measures reflect different levels of biology – enzyme assays provide information about activity that remains under the assay conditions at the time of sampling, while PICRUSt2 provides a prediction of the functional repertoire that might be derived from the 16S rRNA profile. This divergence may be due to exhaustion of easily hydrolysable substrates and a decrease in extracellular depolymerization activity, whereas the community maintains or increases the genetic potential for downstream nitrogen transformations. This is in line with the general rule that community composition and putative functional potential are not necessarily correlated with measured process rates (Douglas et al., 2020; Pérez-Losada et al., 2022). Substrate availability, sorption and environmental conditions also affect the extracellular enzyme activities and a decrease at the end point is not interpreted as a general decrease of the nitrogen turnover capacity.

There are two analytical boundaries of significance. First, PICRUSt2 predictions are not measurements of gene copy number, transcription or enzyme flux (Douglas et al., 2020). Second, patterns of correlation in genus-EC and ASV networks do not necessarily mean that organisms are directly interacting; rather, a similar pattern could be observed if the organisms share the same response to factors such as pH, oxygen, moisture or substrate availability. A positive correlation (*r* = 0.68) between predicted N-cycle functions and inorganic N accumulation also provides ecological coherence to the inference but again does not rule out these alternatives. The developed model should therefore be considered a collection of mechanistic hypotheses with taxa as the first choice for validation, followed by pathways and finally time windows. A critical follow-up should measure functional genes and transcripts of ammonification, nitrification and denitrification in addition to inorganic-N pools and gaseous N losses as a function of time. Stable-isotope tracing could then be used to resolve taxa-pathway coupling for actual instead of only potential nitrogen fluxes.

### 4.5 Implications and next steps for SMS valorization

Earlier studies have demonstrated that vermicomposting can enhance organic residues quality and earthworms can modify the microbial community and extracellular activity (Gong et al., 2019; Yu et al., 2022). The current study provides a temporal, substrate-resolved perspective, by simultaneously considering physicochemical trajectories, bacterial succession, predicted nitrogen cycle functions, and network structure. Synthesis results refine a simple acceleration model of vermicomposting. In addition to a more positive inorganic-N accumulation, earthworms were linked to a reorganization of community composition and co-occurrence patterns. This interpretation is supported by the composting studies showing that nitrogen-cycling functional profiles and microbial associations change together under management conditions (Wang et al., 2022). More broadly, microbial community shifts in response to experimental environmental perturbation can also alter N pathways (e.g., nitrification and denitrification), and the direction and magnitude of the change are system dependent (Seeley et al., 2020). The current data thus point to a shift in the ecological niche associated with earthworms, but not a general microbial signature that could be associated with nitrogen mineralization.

From an applied perspective, these data identify substrate blending and earthworm-associated habitat modification as testable levers for SMS valorization. The present C/N data support considering T3 as one candidate blend, but do not establish its superiority or final product quality. The experiment was designed to characterize physicochemical and microbial dynamics rather than certify the final compost product. Accordingly, maturity, heavy metal content and phytotoxicity were outside the predefined analytical scope and were not measured. Its suitability should therefore be tested at pilot scale using replicated measurements of maturity, heavy metal content, phytotoxicity, pathogen safety, nutrient release and nitrogen loss. Similarly, *Mesorhizobium* and *Flavobacterium* are promising targets for targeted isolation or enrichment studies, but they are not validated inoculant strains. Coupling isolate-based assays or synthetic communities with metagenomics and metatranscriptomics or direct process-rate measurements would clarify whether these taxa actively contribute to improve nitrogen retention or if they simply indicate favourable composting conditions. Within this framework, the microbial profile would be used to monitor the process, and subsequently, following causal validation, as a target for intervention. This validation is needed before the community reassembly can be considered a controllable engineering parameter for the conversion of the SMS to a stable nitrogen-enriched organic fertilizer.

## 5 Conclusion

*E. fetida*-mediated composting was associated with higher inorganic-N concentrations in SMS, restructuring of the bacterial community, shifts in predicted nitrogen-cycle functions and alteration of microbial network structure. Candidate genera associated with the observed patterns included *Arenimonas*, *Pseudoxanthomonas*, *Croceibacterium* and *Kineobacterium*. *Mesorhizobium* and *Flavobacterium* were also at the core of the association networks inferred. These shifts coincided with marked increases in NH_4_^+_^N and NO_3_^−_^N, consistent with higher measured inorganic-N concentrations. The results indicate that earthworm composting is a candidate approach for SMS valorization, but they do not establish total nitrogen retention or process-rate enhancement. The proposed functional mechanisms are based on 16S-derived predictions and network associations and require direct validation by multi-omics and targeted functional assays.

## Data Availability

The original contributions presented in the study are publicly available. This data can be found at the National Center for Biotechnology Information (NCBI) using accession number PRJNA1405922.
